# Design of Fluorescent Hybrid Materials Based on POSS for Sensing Applications

**DOI:** 10.3390/molecules27103137

**Published:** 2022-05-13

**Authors:** Sha Ding, Shuai Zhao, Xingyue Gan, Aokui Sun, Yong Xia, Yuejun Liu

**Affiliations:** 1 Key Laboratory of Advanced Packaging Materials and Technology of Hunan Province, Hunan University of Technology, Zhuzhou 412007, China; 421298314@163.com (S.D.); 17729073093@163.com (S.Z.); 17734655199@163.com (X.G.); aksun@hut.edu.cn (A.S.); 2 College of Chemistry and Chemical Engineering, Central South University, Changsha 410083, China

**Keywords:** polyhedral oligomeric silsesquioxane, hybrid materials, fluorescence, chemo-sensor

## Abstract

Polyhedral oligomeric silsesquioxane (POSS) has a nanoscale silicon core and eight organic functional groups on the surface, with sizes from 0.7 to 1.5 nm. The three-dimensional nanostructures of POSS can be used to build all types of hybrid materials with specific performance and controllable nanostructures. The applications of POSS-based fluorescent materials have spread across various fields. In particular, the employment of POSS-based fluorescent materials in sensing application can achieve high sensitivity, selectivity, and stability. As a result, POSS-based fluorescent materials are attracting increasing attention due to their fascinating vistas, including unique structural features, easy fabrication, and tunable optical properties by molecular design. Here, we summarize the current available POSS-based fluorescent materials from design to sensing applications. In the design section, we introduce synthetic strategies and structures of the functionalized POSS-based fluorescent materials, as well as photophysical properties. In the application section, the typical POSS-based fluorescent materials used for the detection of various target objects are summarized with selected examples to elaborate on their wide applications.

## 1. Introduction

Fluorescent hybrid materials, which combine the advantages of both organic and inorganic material, have become one of the most popular star materials in modern society due to their potential applications in many fields such as sensor technology [1,2,3,4,5,6], photoelectric device [7,8,9,10,11,12], biomedicine [13,14,15,16,17], and environmental sciences [18,19,20,21]. However, the demand for high-performance and multifunctional fluorescent materials has increased dramatically along with the development of science and technology. Therefore, researchers have been working on the design and development of high-performance materials to expand application fields. 

Among many hybrid materials, polyhedral oligomeric silsesquioxane (POSS) is favored by researchers because of its nanoscale size, well-defined framework, thermostability, low toxicity, biocompatibility, as well as customizable properties by conventional physical or chemical techniques. POSS is classified as hybrid inorganic-organic materials with the empirical formula (RSiO_1.5_)_n_, in which R can represent the great variety of organic substituents, while n is commonly 6, 8, 10, or 12. The cage-like octameric structures (*n* = 8) are among the most promising precursors for further development [22,23,24,25,26], and are most widely used in various fields [27,28,29,30,31,32]. Thus, in this review, the POSS specifically refers to its structure containing cage-like (RSiO_1.5_)_8_, unless otherwise specified. In these compounds, the cubic inorganic cores consisting of eight silicon corner atoms and twelve oxygen edge atoms (Si-O-Si) are mono-dispersed (0.53 nm in diameter), while the eight organic arms (R) attached to the eight silicon corner atoms are distributed in three dimensions. The rigid cubic inorganic cores give them well-defined nanoscale size, while the R substituents give them tailorable chemical and physical properties. In the last three decades, the field of POSS has developed greatly due to its well-defined nanostructures, facile chemical modification, and inexpensive precursor materials. There are a number of reviews on the synthesis of POSS compounds [22,33,34,35], and applications of POSS-based polymeric materials in drug delivery, photodynamic therapy, and bioimaging [36,37,38,39,40,41,42,43,44,45]. However, we found no review on the advancement of fluorescent hybrid materials based on POSS, describing their properties and applications. In this review, we will mainly focus on the recent progress in the design of POSS-based fluorescent materials and their sensing applications. 

## 2. Synthesis and Design of POSS-Based Fluorescent Materials

The incorporation of POSS into fluorescent materials can greatly improve the performance of the materials and give them more functionality. The preparation of POSS-based fluorescent materials can be divided into chemical synthesis and physical blending. Recently, a variety of the traditional and the burgeoning chemical synthesis methods have been widely used in the design and synthesis of POSS-based fluorescent materials, such as hydrolytic condensation [46], hydrosilylation [47,48,49,50], Heck reaction [51,52,53,54], atom transfer radical polymerization (ATRP) [55,56,57], reversible addition-fragmentation chain transfer polymerization [58], ring-opening metathesis polymerization [59], as well as “click” chemistry reaction [60,61,62,63]. The method of physical blending is mainly used in the quantum dots (QDs) coated and lanthanide-doped POSS-based fluorescent materials [64,65,66,67].

### 2.1. POSS-Based Organic-Molecule Fluorescent Materials

**Figure 1:**
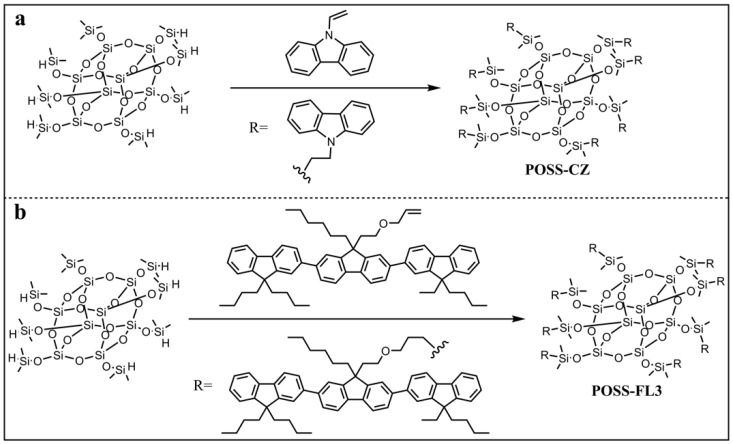
(**a**) Molecular structures of POSS-CZ [68]. (**b**) Molecular structures of POSS-FL3 [69].

Organic small molecule fluorescent materials are favored by researchers because of their simple structure, abundant commercially available raw materials, and easy to modify properties. In 2005, Kawakami and co-workers were the first to introduce the photo- and electroactive π–chromophore into the POSS system by hydrosilylation reaction (Figure 1a) [68]. The structure of cotakis [2 -(carbazol-9-yl)ethyldimethylsiloxy]silsesquioxane (**POSS-Cz**) was characterized by ^1^H, ^13^C, and ^29^Si NMR, IR, and MALDI-TOF MS spectroscopies. **POSS-Cz** had good solubility in general organic solvents, such as THF, toluene, dichloromethane, and chloroform. The solid state emission showed a strong monomeric emission peak with quantum yield *Ф* = 0.27 (refer to *p*-terphenyl *Ф* = 0.87), which indicated the suppression of the formation of excimers even in the solid state. It provides a feasible idea for the development of novel photo- and electroactive materials. Shim and co-workers synthesized a POSS-based blue light electroluminescent nanoparticle (**POSS-FL3**) by the hydrosilation reaction between octakis(dimethylsiloxy)silsesquioxane and an allylfunctionalized terfluorene chromophore (Figure 1b) [69]. The maximum absorption of **POSS-FL3** was found to be 352 nm in THF, while the maxima of the fluorescence emission were at 394 and 415 nm. Because of the good spectral overlap of the maximum absorption wavelength (391 nm) of poly(dihexylfluorene) with the maximum emission wavelengths (394 nm) of **POSS-FL3**, **POSS-FL3** could be used as a nanoparticle-cored dopant chromophore to enhance the quantum efficiency of poly(dihexylfluorene) through energy transfer.

**Figure 2:**
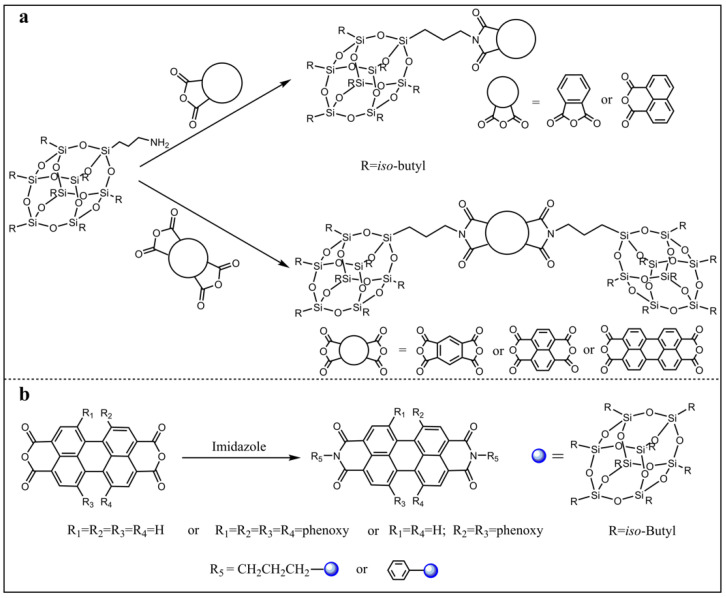
(**a**) Synthetic procedure and molecular structures of a variety of POSS imides [70]. (**b**) Synthetic procedure and molecular structures of a series of dumbbell POSS-PDI [71].

Clarke and co-workers synthesized a variety of POSS imides by condensation of amine-POSS with a variety of mono- and bis-anhydrides (Figure 2a) [70]. Their structures were characterized by FIRT, ^1^H, ^13^C, and ^29^Si NMR, and ESI mass spectrometry. The naphthyl POSS mono/bisimide showed extremely weak fluorescence, while the perylene POSS bis-imide exhibited very strong fluorescence with a quantum yield approaching unity. The single crystal X-ray crystallography indicated that the bulky POSS partially isolated the perylene moieties and thus reduced the aggregation of the fluorophore in the solid state. Subsequently, Li and co-workers synthesized a series of dumbbell **POSS-PDI** (POSS-perylene diimide) triads by the same method (Figure 2b) [71]. The photophysical properties of these compounds both in the solution and solid state were studied by UV-vis absorption spectroscopy, fluorescence spectroscopy, and fluorescence quantum yield measurement. The spectroscopic properties of these compounds were infinitesimally affected by the POSS groups in the solution, while there was a great influence in the solid state. In the solid state, the bulky POSS groups changed the packing structure of the fluorophores, thus affecting the solid state emission properties. However, the solid state fluorescence quantum yield, which is determined by the packing structure of the fluorophores, did not increase inevitably as expected. The large longitudinal displacement resulted in the “J” aggregation form and increase of fluorescence quantum yields, while the small longitudinal displacement caused “H” aggregation form and small fluorescence quantum yields.

**Figure 3:**
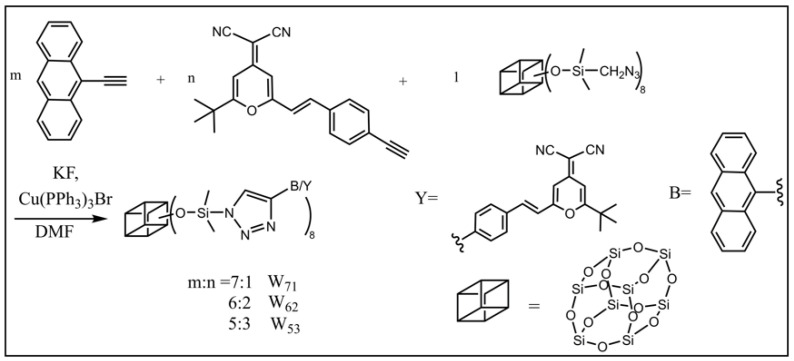
The synthetic route of the POSS-WLED [72].

Xu and co-workers reported a POSS-based white-light-emitting single molecular nano-hybrid (**POSS-WLED**) vis a facile “Azide-acetylene” click chemistry reaction by simply controlling the feed ratio of blue (9-ethynyl anthracene, mark as **B**) and yellow (2-(2-tert-butyl-6-(4-alkynyl styrene)-4H-4-sub pyranyl) two propylene nitrile, mark as **Y**) emitting units (Figure 3) [72]. The optimized molecular structure and composition of **POSS-WLED**, in which the component ratio of **B**:**Y** is 6:2 (defined as **W_62_**), were obtained by theoretical simulations and molecular design. The introduction of nano-sized POSS not only showed a significant aggregation-induced enhancement effect and decoupling effect of the emitter, but also exhibited high thermal stability and a significantly enhanced fluorescence emission with *Ф* = 0.95 in the solid film. This work provided a strategy for the design and preparation of single white-light-emitting molecules with high thermal stability and emission efficiency.

### 2.2. POSS-Based Polymer Fluorescent Materials

Organic fluorophores have high fluorescence efficiency in solution, but its luminescence intensity will be greatly weakened in the condensed state. The unique cage-like nanostructure of POSS can effectively inhibit intermolecular aggregation and enhance fluorescence efficiency, thus it is widely used in the design of various organic fluorescent materials including fluorescent polymers. The introduction of POSS into conventional fluorescent polymers can not only improve the condensed fluorescent efficiency, but also endow fluorescent materials with some excellent special properties to expand their applications.

**Figure 4:**
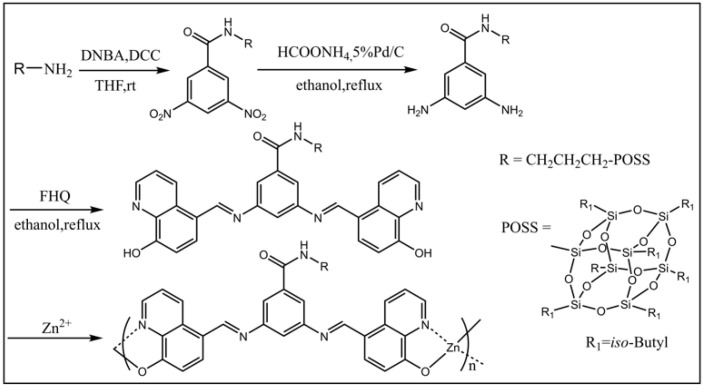
Synthetic procedure of the ligands and polymers [73].

**Figure 5:**
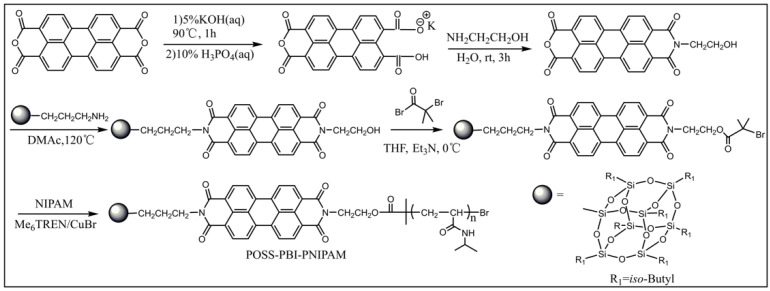
Synthetic procedure of the PBI-bridged amphiphilic polymer POSS-PBI-PNIPAM [74].

**Figure 6:**
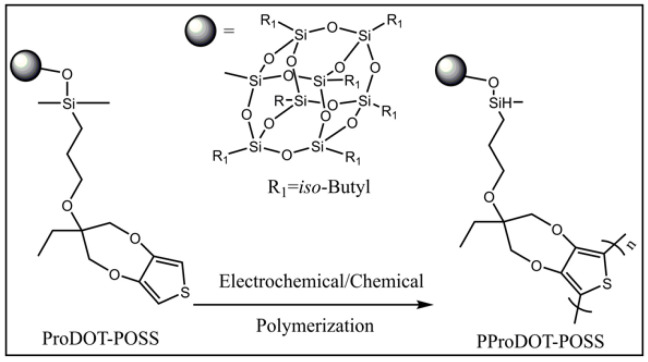
Synthetic procedure of PProDOT-POSS [75].

Bai and co-workers synthesized a novel bis(8-hydroxyquinoline) zinc-based conjugated coordination polymer with POSS in the side chains by the dehydration condensation reaction [73], which was the first example of the introduction of POSS units into the conjugated coordination copolymers to design solid state fluorescent materials, shown in Figure 4. The introduction of nano-sized POSS not only effectively improved the solubility of the coordination polymers, but also prevented the fluorescence quenching effect caused by intermolecular aggregation in the solid state. Compared to the polymer containing dodecyl (*Ф* = 0.02), the polymer containing POSS showed a strong fluorescence at 613 nm with a much higher quantum yield *Ф* = 0.26 in the solid state. The green fluorescent nanoparticles with excellent stability, which showed strong fluorescence at 545 nm, were obtained by facile self-assembly of POSS-based fluorescent polymers in chloroform solution. Subsequently, they designed and synthesized a novel amphiphilic polymer in possession of a perylene diimide bridge between a POSS unit and a PNIPAM chain by combining condensation and ATRP reactions (Figure 5) [74]. The intermediate and the target polymer were characterized by NMR, FT-IR, elemental analyses, and GPC. Hybrid fluorescent nanoparticles were prepared by the self-assembly of the polymers in aqueous solution. The hybrid fluorescent nanoparticles retained the luminescence in its aggregate state and showed red emission at 645 nm with the quantum yield *Ф* = 0.27. The fluorescence intensity of the hybrid fluorescent nanoparticles could be adjusted by changing the temperature. The red hybrid fluorescent nanoparticles with thermos-responsive had potential applications in biosensors and drug delivery. Cihaner and co-workers synthesized the alkyl-substituted POSS integrated poly(3,4-propylenedioxythiophene) conjugated polymer **PProDOT-POSS** via both chemical and electrochemical polymerization methods (Figure 6) [75]. The corresponding polymer was soluble completely in common organic solvents such as toluene, dichloromethane, and chloroform. It showed a red/orange emission at 605 nm in toluene when excited at 500 nm. The optical bandgap of its neutral film with a maximum absorption band at 555 nm was calculated as 1.95 eV. The results indicated that the **PProDOT-POSS** could be used as promising candidates for optoelectronic and bioelectronics applications.

### 2.3. POSS-Based QDs Fluorescent Materials

**Figure 7:**
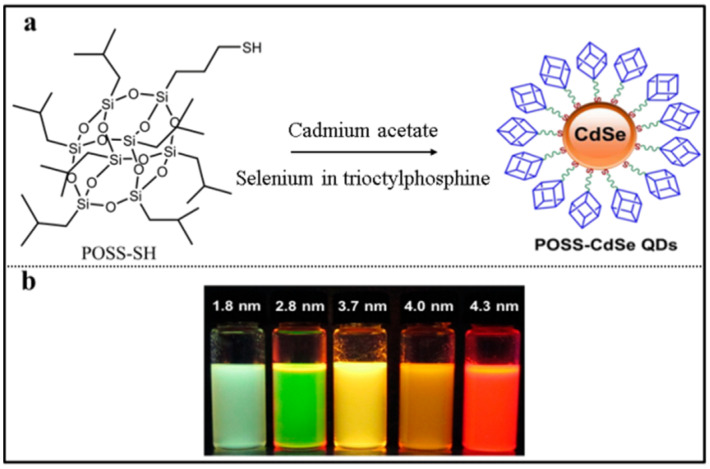
(**a**) Synthetic procedure of POSS-CdSe QDs [80]. (**b**) The photoluminescence color change of the POSS-CdSe QDs under a UV lamp during the growth [80].

**Figure 8:**
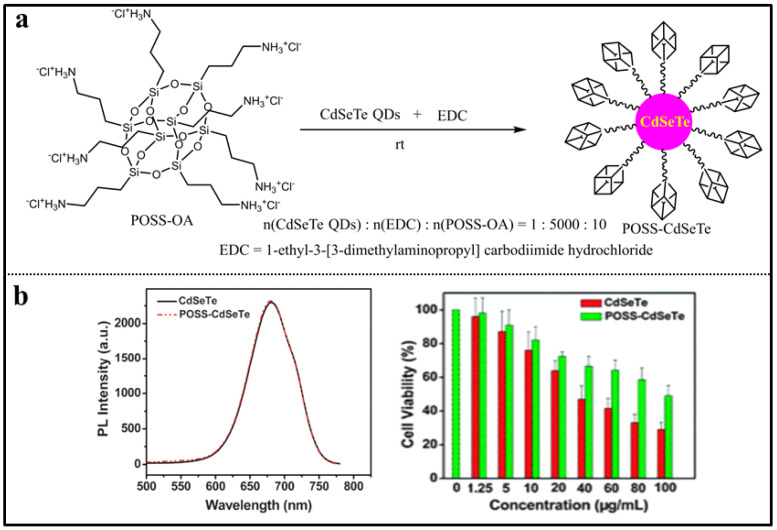
(**a**) Synthetic procedure of POSS-CdSeTe QDs [81]. (**b**) The PL spectra and cytotoxicity of SiHa cells incubated with different concentrations of POSS-CdSeTe QDs and CdSeTe QDs [81].

Quantum dots (QDs) are fluorescent semiconductor nanoparticles composed of groups II–VI or III–V elements with a diameter of 2–10 nm [76,77,78]. QDs have unique optical and electronic properties due to the quantum confinement effects. However, the practical applications of QDs are impeded by their water solubility and toxicity. The development and search for novel ligands for QDs providing them desirable functionalities constitutes a hot research field nowadays [79]. In 2013, the POSS-based CdSe quantum dots, denoted as **POSS-CdSe QDs**, were reported by Rogach and co-workers (Figure 7a) [80]. They synthesized the QDs using a mercapto-substituted polyhedral oligomeric silsesquioxane (POSS-SH) as ligand. Due to the bulkiness of the siloxane core, the **POSS-CdSe QDs** had adjustable particle size and improved light-emission characteristics (Figure 7b). The absorption and photoluminescence (PL) spectra were used to monitor the growth kinetics of **POSS-CdSe QDs** in chloroform. After 2 min of growth, the size of particles was 1.8 nm with a characteristic absorption maximum at 430 nm and a broad PL spectrum (near-white light emission). The absorption maximum shifted to 527 nm after 3 min of growth with the size of particles 2.8 nm, the narrow PL peak in the green spectral range. After 10 min of growth, the size of particles was 3.7 nm with the emission color of the growing samples gradually changed from green to red. Subsequently, low toxicity near-infrared-emitting QDs were fabricated in aqueous medium by conjugating octa-aminopropyl polyhedral oligomeric silsesquioxane (OA-POSS) to CdSeTe QDs through condensation reaction, namely **POSS-CdSeTe QDs** (Figure 8a) [81]. The **POSS-CdSeTe QDs** had improved biocompatibility and retained the unique photophysical properties of CdSeTe quantum dots, in which the PL peak reached 681 nm with a quantum yield of 0.26 (Figure 8b). The toxicity of CdSeTe quantum dots to living cells was significantly reduced by covering the surface with OA-POSS. These results indicated that **POSS-CdSeTe QDs** were very promising fluorescent labels in the biomedical field.

**Figure 9:**
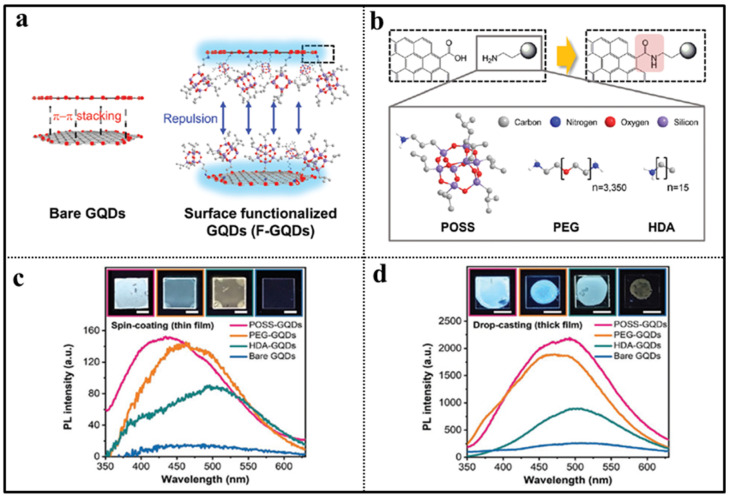
(**a**) Schematic diagram of inhibited fluorescence quenching of surface-functionalized graphene quantum dots (F-GQDs). The fluorescence quenching of bare GQDs without functionalization due to π−π stacking [83]. (**b**) Synthetic procedure of F-GQDs (POSS-GQDs, PEG-GQDs, and HDA-GQDs) [83]. (**c**) PL spectra of F-GQDs and bare GQDs in spin-coated film [83]. (**d**) PL spectra of F-GQDs and bare GQDs in drop-casted film [83].

Graphene quantum dots (GQDs) [82], as nanometer-sized graphene derivatives with low toxicity, low-cost processing, and unique PL properties, provide new possibility to replace the aforementioned QDs due to their high PL stability. However, the strong π–π stacking interactions induce the aggregation-caused PL quenching (ACQ) in the solid state, which greatly limits their application. Recently, Jeon and co-workers demonstrated for the first time that surface functionalization of QDs (F-GQDs) by POSS, poly(ethylene glycol) (PEG), and hexadecylamine (HDA) could effectively reduce ACQ (Figure 9) [83]. The surface functionalization was achieved by the carbodiimide coupling chemistry between the primary amine of POSS, PEG, and HDA with the carboxyl groups on the edge of the GQDs. In solutions, the photophysical properties of F-GQDs were similar to those of bare GQDs with the absorption peak at less than 300 nm and PL peak at about 400 nm. While the POSS-GQDs, PEG-GQDs, and HDA-GQDs showed a significant enhancement in PL intensity compared to bare GQDs by 9.5-, 9.0-, and 5.6-fold in spin-coated film form and by 8.3-, 7.2-, and 3.4-fold in drop-casted film form, respectively. The results indicated that the POSS was an excellent functionalizing reagent for GQDs.

### 2.4. POSS-Based Lanthanide Fluorescent Materials

**Figure 10:**
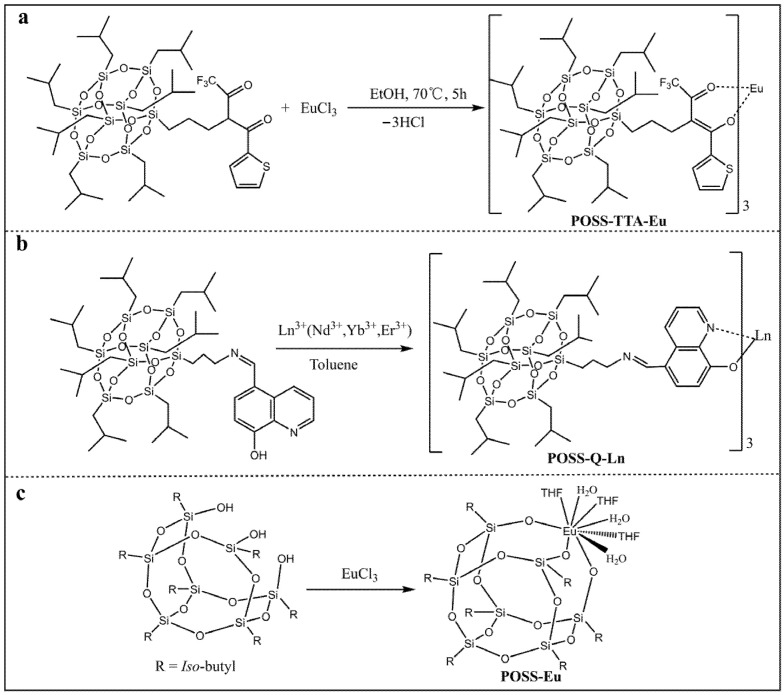
(**a**) Synthetic procedure of POSS-TTA-Eu [86]. (**b**) Synthetic procedure of POSS-Q-Ln [87]. (**c**) Synthetic procedure of POSS-Eu [88].

Lanthanide fluorescent materials are widely used in various fields due to the fascinating optical properties such as sharp emission, high quantum yield, large stock’s shifts, good optical stability, and low toxicity [84]. Unfortunately, the practical applications of the lanthanide fluorescent materials are severely impeded by their inherent drawbacks such as low thermal stability, poor mechanic properties, as well as the tendency to aggregate [85]. Li and coworkers reported a europium (Ⅲ) β-diketonate complex functionalized POSS by the complexation of Eu^3+^ ions with thenoyltrifluoroacetone functionalized POSS for the first time, namely **POSS-TTA-Eu** (Figure 10a) [86]. The as-prepared fluorescent material was a viscous liquid at room temperature, and showed a bright-red emission with long lifetime, high color purity, as well as good thermal stability and processibility. Recently, Li and coworkers used a similar method to synthesize a series of lanthanide (Er^3+^, Yb^3+^, and Nd^3+^) 8-hydroxyquinoline complex-functionalized POSS, namely **POSS-Q-Ln** (Figure 10b) [87]. Compared with the POSS-free lanthanide complexes, the **POSS-Q-Ln** showed obviously enhanced fluorescence emission intensity, which was mainly due to the steric-hindrance effects of the POSS moiety in the complexes. In contrast, Boccaleri and co-workers synthesized a novel fluorescent europium (Ⅲ)-containing POSS (denoted as **POSS-Eu**), in which the europium ion existed in the cage-like structure (Figure 10c) [88]. The Eu^3+^ ion caped the POSS core by the dangling oxygen groups of the open corner and water and tetrahydrofuran molecules in the reaction medium, which was confirmed by NMR, FT-IR, and MALDI-TOF analyses.

## 3. Sensing Application of POSS-Based Fluorescent Materials

### 3.1. Metal Ion Sensing

**Figure 11:**
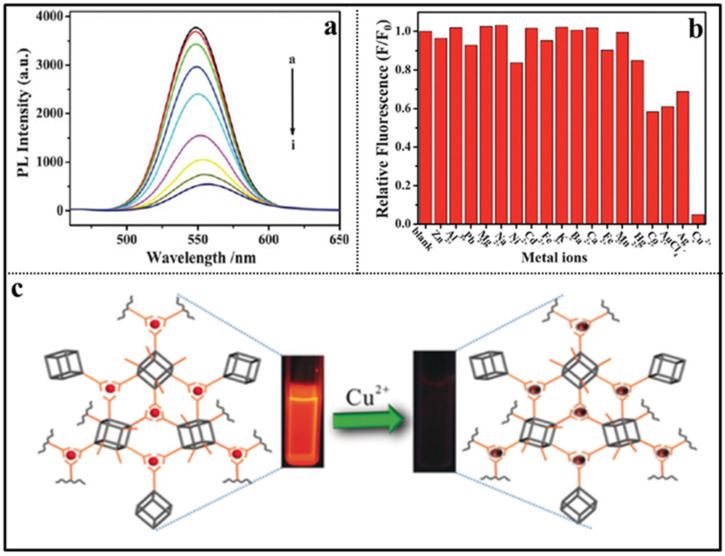
(**a**) Effect of Cu^2+^ on the luminescence of the POSS-CdTe QDs at pH 7.4. The concentrations of Cu^2+^ (μmol/L) in (a–i) are 0, 0.05, 0.1, 0.2, 0.4, 0.6, 0.8, 1 [65]. (**b**) Quenching effect of different ions on the fluorescence intensity of the POSS-CdTe QDs in a PBS buffer solution at pH 7.4 [65]. (**c**) Schematic diagram of the sensing process of POSS-Eu(dpa)_3_ toward Cu^2+^ [89].

**Figure 12:**
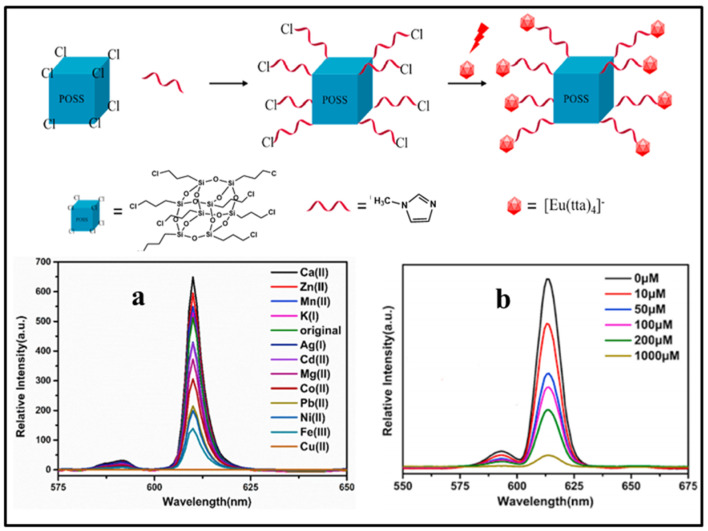
Synthetic procedure of POSS-min- [Eu(tta)_4_]. The PL spectra of POSS-min- [Eu(tta)_4_] to different aqueous solution of metal cations (**a**) and to different concentration aqueous solution of Cu^2+^ (**b**) [90].

**Figure 13:**
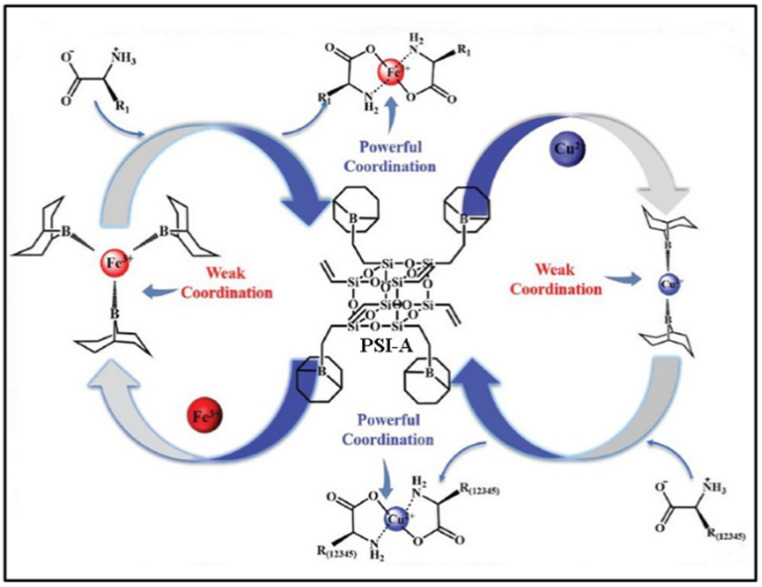
Schematic illustration of sensing mechanism of PSI-A towards Cu^2+^, Fe^3+^, and amino acids [91].

The identification and detection of metal ions are very important for monitoring environmental pollution, food safety, and human health. Copper is one of the earliest metals used by humans and an essential trace element in many living biological systems, but an excess of Cu^2+^ concentration may cause environmental contamination and biological toxicity. It is extremely important to detect the concentration of Cu^2+^ in environmental and biological samples with high selectivity and sensitivity. In 2013, Hao and coworkers reported a POSS-coated CdTe QDs fluorescent sensor for detecting Cu^2+^ in aqueous medium based on selectively fluorescence quenching (ON-OFF) [65], as shown in Figure 11. Upon addition of Cu^2+^, the fluorescence intensity of the POSS-CdTe QDs sensor was obviously quenched due to Cu^2+^ ions binding on the surface of the sensor (Figure 11a). The selective fluorescence quenching experiment indicated that the sensor had more sensitivity to Cu^2+^ than to Ni^2+^, Co^2+^, Ag^+^, and little effect on the other cations (Na^+^, Ca^2+^, Mn^2+^, Hg^2+^, Mg^2+^, K^+^, Al^3+^, and Zn^2+^) in the PBS buffer solution at pH 7.4 (Figure 11b). Later, a similar turn-off fluorescent material based on POSS-Eu(dpa)_3_ was reported by Li and co-workers (Figure 11c) [89]. Compared to other common metal ions, the as-prepared fluorescent material presented an effective fluorescence quenched for Cu^2+^ in aqueous media with the largest *K*_sv_ value of 2359.6 M^−1^ (based on the Stern–Volmer equation). Recently, Li and co-workers reported a ionic liquid functionalized POSS fluorescent sensor (POSS-min-[Eu(tta)_4_]) that had selective fluorescent quenching effect for Cu^2+^ with fast response, high sensitivity and selectivity (Figure 12) [90]. Due to the strong effect of the exchange interaction between Cu^2+^ and Eu^3+^ of POSS-min- [Eu(tta)_4_], the *K*_sv_ value for Cu^2+^ was 17,160 M^−1^. The detection limit of POSS-min- [Eu(tta)_4_] (based on the standard of IUPAC LOD = 3σ/k) for Cu^2+^ in water is 0.011 μM with detection interval ranging from 0 to 1000 μM. Furthermore, Zuo and co-workers reported a new “ON-OFF-ON” type POSS-based fluorescent probe (denoted as PSI-A) for the reversible detection of Cu^2+^, Fe^3+^, and amino acids (Figure 13) [91]. The fluorescence emission of PSI-A was dramatically quenched by adding Cu^2+^ and Fe^3+^, while the emission recovered by adding different kinks of amino acids because of the weak coordination bond between Cu^2+^ and Fe^3+^ and PSI-A. The detection limit of PSI-A for Cu^2+^ and Fe^3+^ was approximately 0.0019 μM and 0.0032 μM, respectively.

**Figure 14:**
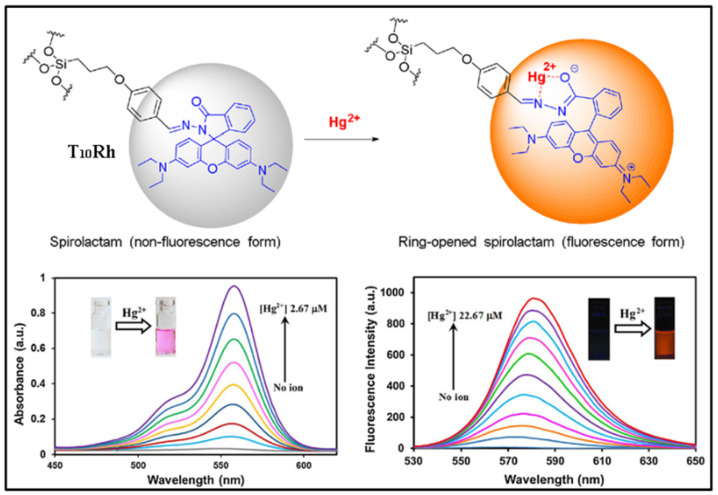
Schematic illustration of sensing mechanism of T_10_Rh toward Hg^2+^. The absorption spectra (**bottom left**) and PL spectra (**bottom right**) of T_10_Rh to different concentration 10% aqueous ethanol solutions of Hg^2+^ [92].

**Figure 15:**
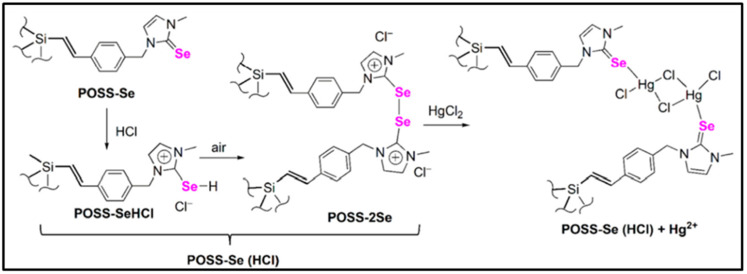
The possible mechanism of detecting Hg^2+^ by POSS-Se after treating with HCl [93].

**Figure 16:**
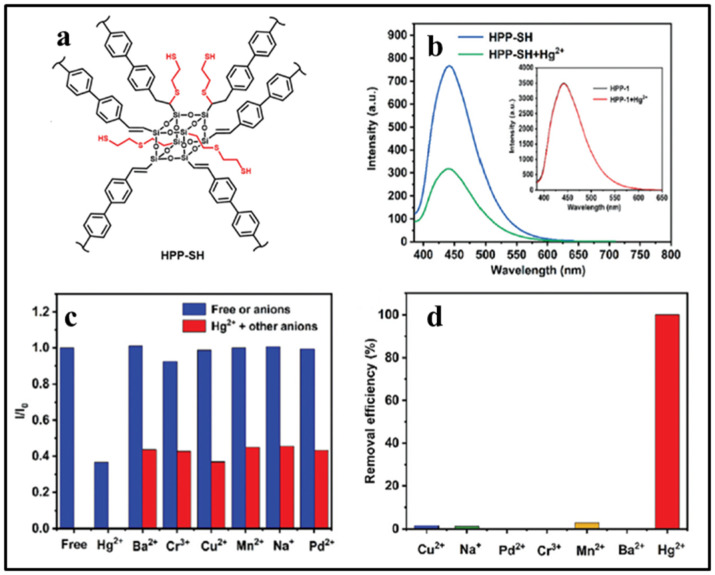
(**a**) Molecular structures of HPP-SH [94]; (**b**) the PL spectra of the HPP-SH suspension (0.1 mg/mL) in 50% aqueous ethanol in the presence and absence of Hg^2+^ (100 ppm) [94]; (**c**) selectivity of the HPP-SH suspension (0.1 mg/mL) for Hg^2+^ [94]; (**d**) the removal efficiency of HPP-SH suspension (1 mg/mL) for Hg^2+^ in the presence of various metal ions (50 ppm) [94].

Compared to other metal ions, mercury ion (Hg^2+^) possesses much more serious toxicity and causes more widespread environmental pollution. New fluorescent sensors with high selectivity and sensitivity for Hg^2+^ have drawn extensive research attention. In 2018, Ervithayasuporn and co-workers reported a dual-mode optical sensor based on rhodamine functionalized POSS for Hg^2+^ [92], namely T_10_Rh. Upon interacting with Hg^2+^, the color of T_10_Rh in the 10% aqueous ethanol solutions changed from colorless to pink-red, and the intensity of the fluorescence emission was greatly enhanced with the emission peak at 580 nm (OFF-ON), as shown in the Figure 14. The detection limit of T_10_Rh for Hg^2+^ was approximately 0.00314 μM. Subsequently, a novel selenone-functionalized polyhedral oligomeric silsesquioxane (POSS-Se) for selective detection and adsorption of Hg^2+^ in aqueous solutions was reported by Feng and co-workers (Figure 15) [93]. The addition of Hg^2+^ had little effect on the solution color and emission intensity of POSS-Se, while the POSS-Se after acid treatment (denoted as POSS-Se (HCl)) could be used as a dual-mode optical sensor for Hg^2+^. As the concentration of mercury ions increases, the colors of the POSS-Se (HCl) solution gradually changed from dark-yellow to pale-yellow, and the intensity of fluorescence emission significantly strengthened (OFF-ON). The detection limit of POSS-Se (HCl) for Hg^2+^ was approximately 0.00848 μM. Moreover, the POSS-Se and POSS-Se (HCl) could be used as excellent adsorbent for mercury ions with the maximum adsorption capacity of 952 and 907 mg/g, respectively. Recently, Wang and co-workers reported a dual-function sulfur-containing POSS-based fluorescent polymer material (denoted as HPP-SH) for selective detection and adsorption of Hg^2+^ ions (Figure 16) [94]. Upon the addition of Hg^2+^ ions, the fluorescence intensity of HPP-SH was significantly decreased (ON-OFF) because of the interaction between Hg^2+^ ions and the thioether or thiol groups of HPP-SH. The detection limit of HPP-SH for Hg^2+^ in water was approximately 0.00448 μM. It should be noted that the sulfur content of HPP-SH played an important role in the detection for Hg^2+^ ions. The higher sulfur contents resulted in a lower limit of detection. Similarly, the HPP-SH could be used as an excellent adsorbent for mercury ions with the maximum adsorption capacity of 900.9 mg/g. In addition, if the initial concentration of mercury ions was less than 20 ppm, the removal efficiency was up to 99.99%.

**Figure 17:**
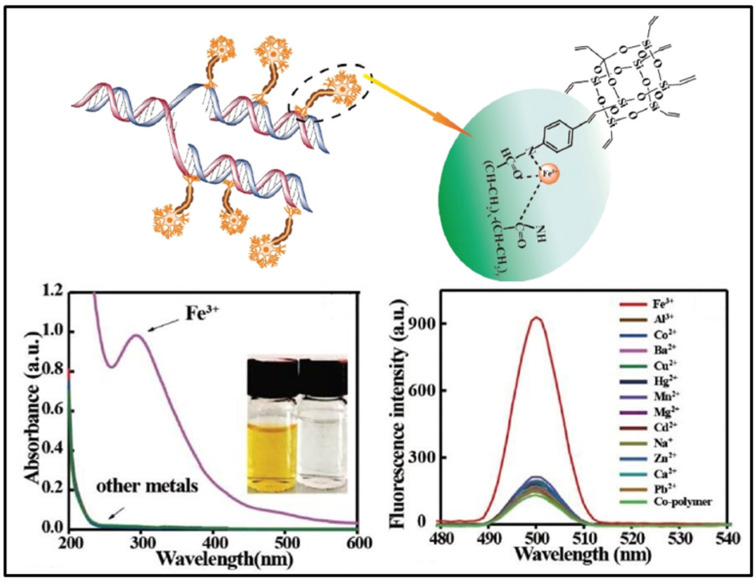
Schematic of the proposed bonding mode between OV-POSS co-poly (acrylamide) and Fe^3+^. The absorption (**bottom left**) and PL (**bottom right**) spectra of OV-POSS co-poly (acrylamide) towards different metal ions in water [95].

Fe^3+^ is one of the essential trace elements to maintain human life and health. In 2019, Xu and coworkers reported a highly selective fluorescence sensor by combining octavinyl-POSS with amine-containing polyacrylamide (denoted as OV-POSS co-poly(acrylamide)) for repetitive detection of Fe^3+^ in pure water (Figure 17) [95]. The sensing mechanism was mainly attributed to the complexation reaction between OV-POSS co-poly(acrylamide) and Fe^3+^. Upon the addition of Fe^3+^ into the OV-POSS co-poly(acrylamide) solution, the fluorescence intensity significantly enhanced, while the other metal ions had no obvious effect. The detection limit of OV-POSS co-poly(acrylamide) for Fe^3+^ was approximately 0.0009 μM. Moreover, compared with other metal ions, Fe^3+^ caused a visible color change to the naked eye from colorless to bright yellow. Feng and coworkers also reported a fluorescence sensor by combining functionalized ionic liquids and POSS (denoted as ILs-POSS) for the detection of Fe^3+^ [96]. The ILs-POSS was used as an “ON-OFF” fluorescent sensor for Fe^3+^ with the detection limit of 0.0791 μM.

**Figure 18:**
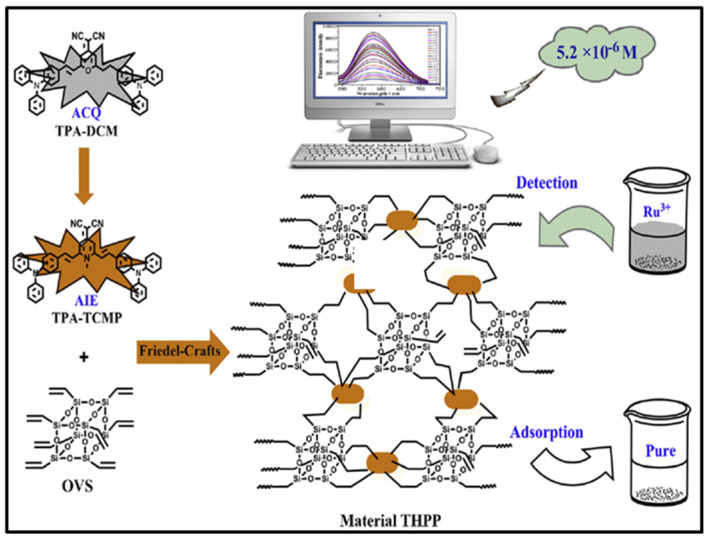
Synthesis of THPP and its application for concurrent detection and adsorption of Ru^3+^ [97].

Nowadays, Ru^3+^ is widely used in catalytic reactions and industrial processes, thus, the rapid detection and efficient removal of Ru^3+^ from water is extremely important to environment protection and human health. Liu and coworkers reported a POSS-based fluorescent nanoporous polymer (denoted as THPP) by Friedel–Grafts reaction of 2-(2,6-bis((E)-4-(diphenylamino)styryl)-1-methylpyridin-4(1H)-ylidene)malononitrile (TPA-TCMP) with octavinyl-POSS for concurrent detection and adsorption of Ru^3+^ (Figure 18) [97]. THPP demonstrated obvious fluorescence ON-OFF sensing for Ru^3+^ ion in DMF/water (5/5, *v*/*v*) solution with the detection limit of 5.2 μM. The THPP could be used as an adsorbent for Ru^3+^ ions with an equilibrium adsorption capacity of 208 mg/g.

### 3.2. Anion Sensing

**Figure 19:**
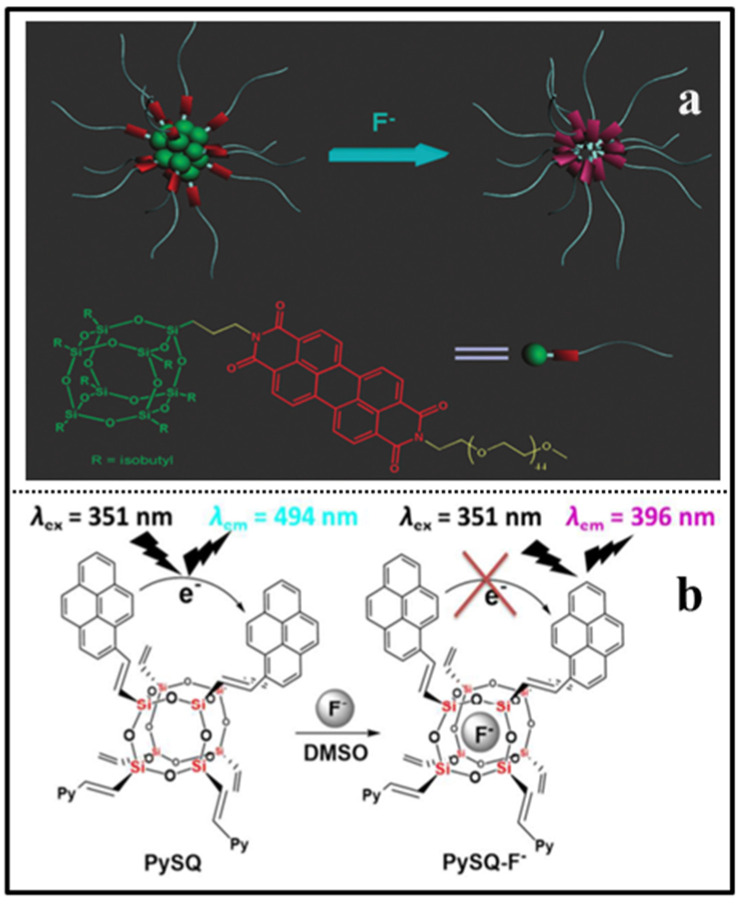
(**a**) Schematic representation of NP structure self-assembled from the POSSPBI-PEO and fluoride ion sensing process in water [98]. (**b**) Schematic representation of PySQ for fluoride ion sensing process in DMSO [27].

**Figure 20:**
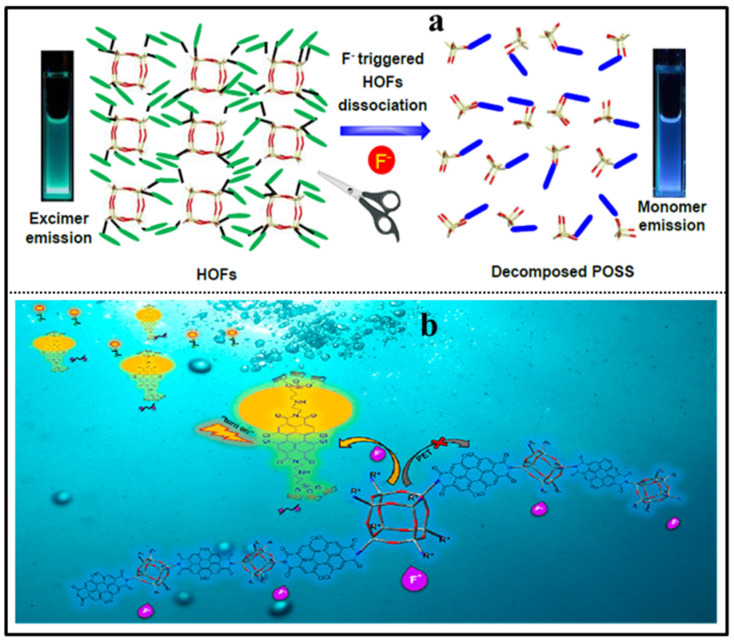
(**a**) Schematic illustration of POSS-based HOFs for the F¯ sensing [100]. (**b**) The possible mechanism of detecting F¯ by AE-PDI [101].

**Figure 21:**
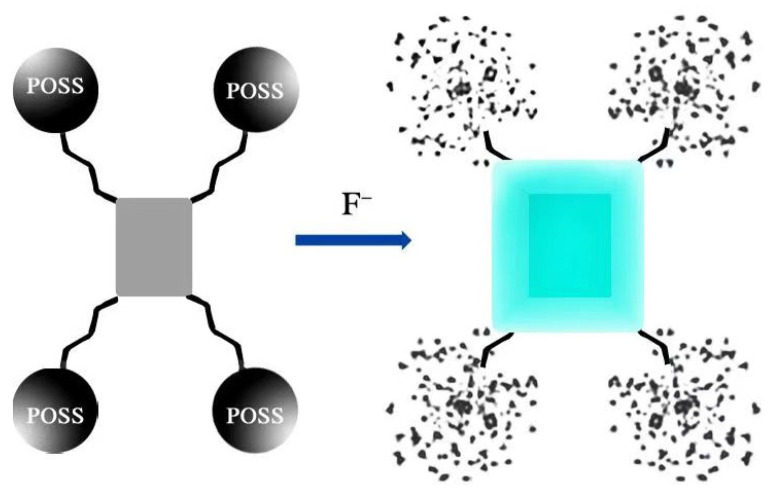
Schematic illustration of the sensing mechanism of TPE-POSS toward F¯ [102].

Fluoride is widely used in dental care and the pharmaceutical industry; however, deficiency or excess of F¯ can cause serious health and environmental problems. The detection of F¯ is undoubtedly important for both human health and environment protection. In 2013, Bai and coworkers reported “ON-OFF” POSS-based red fluorescent nanoparticles (denoted as POSS-PBI-PEO NPs) for the rapid detection of F¯ in water by the fluoride-triggered Si-O bond cleavage mechanism (Figure 19a) [98]. The nanoparticles presented a strong excimer-like emission at 660 nm with a fluorescence quantum yield of 0.26 at room temperature. Upon the addition of F¯ into the POSS-PBI-PEO solution, the fluorescence intensity significantly decreased in less than 10 s with the detection limit of 10 μM, while the other anions had no obvious effect. The selective sensing was due to the specific reaction between F¯ and Si-O bond of the POSS, the decomposition of the POSS cages in the particle cores, which in turn induced aggregation of PBIs groups, thus resulting in quenched fluorescence. However, Ervithayasuporn and coworkers reported pyrene functionalized POSS (denoted as PySQ) for the detection of F¯ by encapsulation-induced fluorescent change (Figure 19b) [27]. The PySQ presented different fluorescent properties in different solvents. PySQ possessed a strong excimer-like emission in DMSO, while the monomer emission of the pyrene groups on PySQ in THF was dominant. Upon the addition of F¯ into the PySQ solution, the excimer-like emission and the monomer emission were both diminished in the low polarity solvent THF, while the excimer-like emission was diminished and the monomer emission was enhanced due to the increased pyrene–pyrene distance in the high polarity solvent DMSO. The response time of the PySQ selective capture of fluoride ions was less than 2 min with the detection limit of 0.00161 μM. Recently, another POSS-based fluorescent porous polymer was reported for fluoride sensing and removal by a similar mechanism, in which anthracene and pyrene were used as polyaromatic spacer groups [99]. In 2019, Tang and coworkers reported a ratiometric fluorescence sensor based on octa-pyrene-modified POSS organic framework nanoparticles for the detection of F¯ (Figure 20a) [100]. The as-prepared nanoparticles possessed excimer-like emission (green emission at 489 nm) due to the stacking of pyrene in their hydrogen-bonded organic framework (HOF) structures. The addition of fluoride ions destroyed the cage structure of POSS and in turn dissociated the HOF structure of the nanoparticles, which resulted in monomer emission (blue emission at 377 nm) of the nanoparticles. The response time of the nanoparticles for fluoride ions was within 10 min with the detection limit of 50 μM. An “OFF-ON” POSS-based hydrophilic luminescent polymer (denoted as AE-PDI) for fluoride ion detection in water/DMSO (98/2, *v*/*v*) solution was reported by Lv and coworkers (Figure 20b) [101], in which perylene diimide was used as fluorophore. Due to the intermolecular photo-induced electron transfer (PET) between amino-POSSs and PDI, the fluorescence emission of the AE-PDI polymer was ultra-weak with a maximum peak at 575 nm. The addition of fluoride ions destroyed the cage structure of POSS and induced fluorescent enhancement at 572 nm by nearly 41 times. The fluorescence enhancing accompanied with the color change (wine red to orange) led to high sensitivity in detection of F¯ ion down to the level of 0.85 μM in water/DMSO media. Recently, Ren and coworkers reported a novel “OFF-ON” fluorescent sensor based on tetraphenylethylene derivative tethered with POSS (denoted as TPE-POSS) for the detection of F¯ (Figure 21) [102]. The TPE-POSS possessed aggregation induced fluorescence enhancement (AIE) features due to the typical AIE luminogen TPE. Upon the addition of F¯ into the TPE-POSS solution, the POSS nanoparticles was collapsed, which resulted in the aggregation of TPE cores and further enhanced the intensity of fluorescence. The fluorescence enhancing led to high sensitivity in detection of F¯ ion down to the level of 0.166 μM.

**Figure 22:**
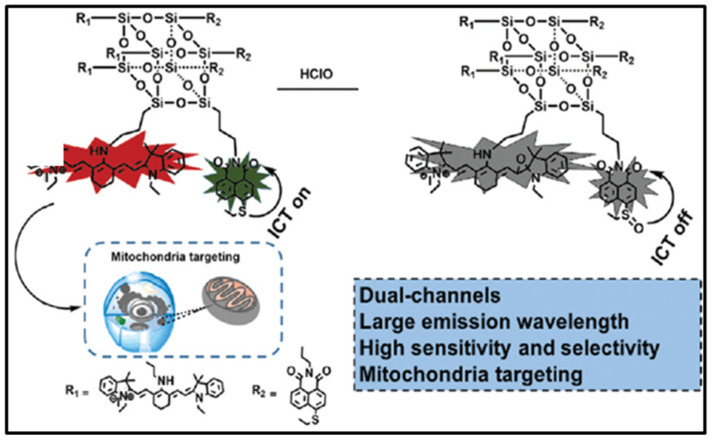
Schematic illustration of the sensing mechanism of POSS-Cy7-N toward ClO¯ [103].

Hypochlorous acid (HClO) plays an important defensive role in the biological immune system, while it may cause many diseases at abnormal concentration. Lin and coworkers reported a dual channels fluorescent probe with both heptamethine cyanine (Cy7) and 1,8-naphthimide based on POSS (denoted as POSS-Cy7-N) for the detection of ClO¯ (Figure 22) [103]. The POSS-Cy7-N possessed a dual emission in the green (510 nm) and the NIR (820 nm) channels. The fluorescence quenching of both channels occurred after the addition of ClO¯ ions, which was due to the disruption of the excited-state (ICT). The POSS-Cy7-N presented high sensitivity in detection of ClO¯ ion down to the level of 0.15 μM.

## 4. Conclusions and Perspectives

This review presents a comprehensive analysis of the design and sensing applications of the POSS-based fluorescent hybrid materials. The different strategies and different types of POSS fluorescent materials were summarized. With the assistance of POSS, the developed fluorescent sensors exhibited high sensitivity, selectivity, and stability for the detection of target object. Although remarkable strides in sensing application were obtained, POSS-based fluorescent hybrid materials still face some challenges in practical application. First, the variety of such fluorescent materials needs to be further enriched. Especially, more high performance chromophores should be developed in POSS-based organic-molecule fluorescent materials. Second, the scope of detection should be further extended, such as explosives, organic pollutant, and so on. Third, the sensing mechanism of POSS-based fluorescent hybrid materials should be further studied. Finally, further strengthening the development of POSS-based fluorescent hybrid materials with both efficient detection and removal functions is conducive to environmental protection.

We anticipate that future research will focus on the development of the following areas, which will benefit the variety and application of POSS-based fluorescent hybrid materials:

(1) Facile and controllable synthesis of POSS-based fluorescent hybrid materials. The “click” chemistry reaction, especially metal-free “thiol-ene” click chemistry, will brilliantly shine in this respect.
(2) Sensing mechanism. Theoretical calculation may be a very effective and practical method to study the sensing mechanism of POSS-based fluorescent hybrid materials by using the density functional theory (DFT) and time-dependent density functional theory (TDDFT).
(3) Sensing application and the others. The detection of explosives and organic small molecular pollutants and the application of fluorescent materials in photodynamic therapy and biological imaging are attracting more and more researchers’ attention. POSS-based fluorescent hybrid materials will make great progress in these areas.

## Data Availability

Not applicable.
